# Vaccination with poly(IC:LC) and peptide-pulsed autologous dendritic cells in patients with pancreatic cancer

**DOI:** 10.1186/s13045-017-0459-2

**Published:** 2017-04-07

**Authors:** Shikhar Mehrotra, Carolyn D. Britten, Steve Chin, Elizabeth Garrett-Mayer, Colleen A. Cloud, Mingli Li, Gina Scurti, Mohamed L. Salem, Michelle H. Nelson, Melanie B. Thomas, Chrystal M. Paulos, Andres M. Salazar, Michael I. Nishimura, Mark P. Rubinstein, Zihai Li, David J. Cole

**Affiliations:** 1 Department of Surgery, Medical University of South Carolina, 96 Jonathan Lucas Street, Charleston, SC 29425 USA; 2 Department of Microbiology and Immunology, Medical University of South Carolina, Charleston, SC 29425 USA; 3 Division of Hematology/Oncology, Department of Medicine, Medical University of South Carolina, Charleston, SC 29425 USA; 4 Departmet of Population Sciences, Hollings Cancer Center, Medical University of South Carolina, Charleston, SC 29425 USA; 5 Department of Surgery, Loyola University Medical Center, Maywood, IL 60153 USA; 6 Center of Excellence in Cancer Research and Zoology Department, Faculty of Science, Tanta University, Tanta, Egypt; 7 Oncovir Inc., 3202 Cleaveland Avenue NW, Washington, DC 20008 USA; 8 Present address: Gibbs Cancer Center and Research Institute, 380 Serpentine Drive, Spartanburg, SC 29303 USA; 9 Present address: Eli Lilly and Company, Lilly Corporate Center, Indianapolis, IN 46285 USA

## Abstract

**Background:**

Dendritic cells (DCs) enhance the quality of anti-tumor immune response in patients with cancer. Thus, we posit that DC-based immunotherapy, in conjunction with toll-like receptor (TLR)-3 agonist poly-ICLC, is a promising approach for harnessing immunity against metastatic or locally advanced unresectable pancreatic cancer (PC).

**Methods:**

We generated autologous DCs from the peripheral blood of HLA-A2^+^ patients with PC. DCs were pulsed with three distinct A2-restricted peptides: 1) human telomerase reverse transcriptase (hTERT, TERT572Y), 2) carcinoembryonic antigen (CEA; Cap1-6D), and 3) survivin (SRV.A2). Patients received four intradermal injections of 1 × 10^7^ peptide-pulsed DC vaccines every 2 weeks (Day 0, 14, 28, and 42). Concurrently, patients received intramuscular administration of Poly-ICLC at 30 μg/Kg on vaccination days (i.e., day 0, 14, 28, and 42), as well as on days 3, 17, 21, 31, 37, and 45. Our key objective was to assess safety and feasibility. The effect of DC vaccination on immune response was measured at each DC injection time point by enumerating the phenotype and function of patient T cells.

**Results:**

Twelve patients underwent apheresis: nine patients with metastatic disease, and three patients with locally advanced unresectable disease. Vaccines were successfully manufactured from all individuals. We found that this treatment was well-tolerated, with the most common symptoms being fatigue and/or self-limiting flu-like symptoms. Among the eight patients who underwent imaging on day 56, four patients experienced stable disease while four patients had disease progression. The median overall survival was 7.7 months. One patient survived for 28 months post leukapheresis. MHC class I –tetramer analysis before and after vaccination revealed effective generation of antigen-specific T cells in three patients with stable disease.

**Conclusion:**

Vaccination with peptide-pulsed DCs in combination with poly-ICLC is safe and induces a measurable tumor specific T cell population in patients with advanced PC.

**Trial registration:**

NCT01410968; Name of registry: clinicaltrials.gov; Date of registration: 08/04/2011).

**Electronic supplementary material:**

The online version of this article (doi:10.1186/s13045-017-0459-2) contains supplementary material, which is available to authorized users.

## Background

Pancreas cancer is currently the 12th most common cancer in the USA [1], yet by 2030, it is expected to become the second leading cause of cancer death [2]. Even when the disease is diagnosed at an early stage, the prognosis is dismal [1]. In metastatic disease, modern chemotherapy regimens such as FOLFIRINOX and nab-paclitaxel plus gemcitabine produce median survival times of less than a year [3, 4], underscoring the urgent need for novel therapies [5]. Despite many agents tested, only the EGFR tyrosine kinase inhibitor erlotinib has gained FDA approval in combination with gemcitabine [6], based on a 2-week improvement in survival compared to gemcitabine alone [7, 8].

In the current era of immunotherapy, a variety of malignancies respond to immune checkpoint inhibitors via activating tumor-reactive T cells [9]. Yet immune checkpoint inhibitors are ineffective in patients with pancreas cancer, perhaps due to the recruitment of immature myeloid cells that overwhelm infiltrating T cells [10, 11]. Vaccines, on the other hand, have the potential to induce an immune response in this setting of “immune privilege” [10]. The most advanced vaccine strategy for pancreas cancer is a combination of low dose cyclophosphamide with GVAX, composed of two irradiated GM-CSF secreting allogeneic pancreas cancer cell lines, followed by CRS-207, a live attenuated *Listeria monocytogenes* that secretes mesothelin [6]. In a landmark phase II study, cyclophosphamide/GVAX prime followed by CRS-207 boost improved overall survival in metastatic pancreas cancer patients compared to cyclophosphamide/GVAX alone [6]. Unfortunately, a subsequent phase 2b trial in third line metastatic pancreas cancer demonstrated a lower overall survival with the GVAX/CRS-207 combination compared to chemotherapy (personal communication), and [12]. So far, the promise of immunotherapy is unfulfilled in pancreas cancer.

One method to induce the antigen-specific CD8^+^ T cell responses in vivo is the use of dendritic cells (DCs) pulsed with antigen [13–15]. DCs pulsed with peptides derived from tumor antigens have shown promise in preclinical models [16]. However, despite inducing the expansion of tumor-reactive T cells in patients, clinical efficacy in cancer patients has been limited [17–19]. Many DC-based adjuvants have been tested in their capacity to activate T cells. Our preclinical studies showed that DCs more effectively augment T cell responses when cultured in presence of poly(I:C), a TLR3 agonist, [20]. In this case, poly(I:C) may act through several mechanisms including the direct activation of DCs. To improve poly(I:C)-mediated therapy, our collaborators developed a GMP-grade stabilized version of poly I:C designated poly-ICLC (Hiltonol®) [21]. Poly(IC:LC) has been evaluated in numerous clinical trials with the goal to boost anti-tumor immunity and was safely administered to patients [22]. Furthermore, in glioblastoma, two studies have shown that the combination of poly(IC:LC) and a DC-based vaccination are well-tolerated [23, 24]. Together, these data suggest that the administration of antigen-pulsed DCs with poly(IC:LC) could expand tumor-reactive T cells in patients with pancreatic cancer.

With the goal of developing combinatorial DC/TLR therapies involving the expansion of tumor-reactive CD8^+^ T cells in patients with pancreatic cancer, this phase I feasibility and safety study was initiated to assess the role of this peptide-pulsed DCs/poly(IC:LC) vaccine in HLA-A2^+^ patients with metastatic or unresectable pancreatic adenocarcinoma. Autologous DCs, prepared from peripheral blood monocytes, were pulsed with three HLA-A2-restricted peptides derived from antigens overexpressed in pancreatic cancer including telomerase, carcinoembryonic antigen (CEA), and survivin [25–27]. The therapy was well tolerated and induced peptide-reactive CD8^+^ T cells. This effort demonstrates the first combination of antigen-pulsed DCs and poly(IC:LC) in patients with pancreatic cancer, and provides a platform for future therapies.

## Methods

### Patient selection

Eligible patients were ≥18 years of age with histologically or cytologically confirmed diagnosis of adenocarcinoma of the pancreas that was metastatic, locally advanced, or recurrent. Patients were required to have HLA-A2 positivity by serological testing Eastern Cooperative Oncology Group performance status ≤2, expected survival >3 months, measureable disease per RECIST 1.1, and adequate organ function [28, 29]. Patients with clinically significant ascites, brain metastases, or HIV were excluded from this trial.

### Study design

**Fig. 1:**
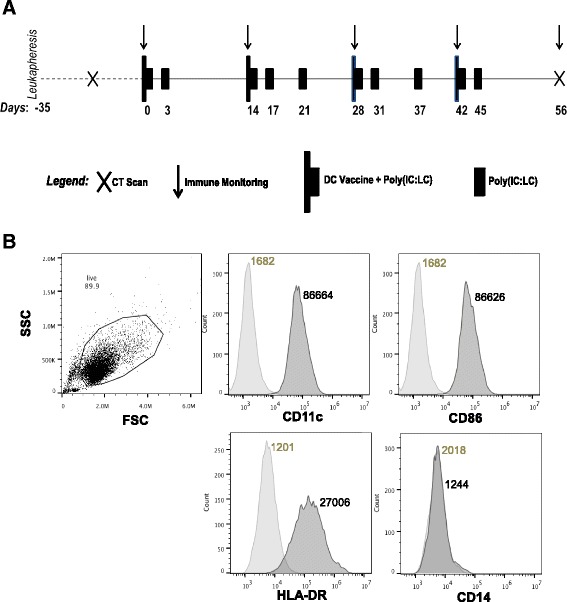
Phenotypic characterization of dendritic cells. **a** Schematic diagram showing the different time points for vaccination and analysis. **b** Dendritic cells (DCs) were prepared from each patient (see Methods). Before treatment administration the DCs were characterized using the flurochrome-conjugated antibodies for cell surface expression of CD11c, CD86, HLA-DR, and CD14. The data was acquired using BD Accuri flow cytometer and analyzed using FlowJo. The numerical values adjacent to the histogram represent the mean fluorescence intensity (MFI)

This pilot study, with a planned sample size of 12 patients, was designed to evaluate the feasibility and the safety of systemic administration of polyinosinic-polycytidylic acid stabilized with polylysine and carboxymethylcellulose (poly(IC:LC), or Hiltonol; Oncovir, Washington, D.C.) concurrent with active vaccination of autologous peptide pulsed DCs in patients with advanced adenocarcinoma of the pancreas. The DC vaccine consisted of a pool of three aliquots of DCs pulsed with hTERT (YLFFYRKSV) [25–27], Cap1-6D (YLSGADLNL) [30], or survivin (LTLGEFLKL) [31, 32] peptides that were obtained from PolyPeptide Group (San Diego, CA). The CEF control peptide pool was obtained from AnaSpec, (cat# 61036, Fremont, CA). The CEF control peptides are 8–12 amino acids in length, with sequences derived from the human *C*ytomegalovirus, *E*pstein-Barr Virus and In*f*luenza Virus. Eligible patients underwent leukapheresis on day -35 to generate immature DCs. At day -28 DCs were cryopreserved and subsequently tested according to lot release criteria. Patients were given a combination vaccine comprised of antigen-pulsed DCs (1 × 10^7^ DC intradermally delivered on days 0, 14, 28, and 42) and TLR3 agonist Poly(IC:LC) (30 μg/kg intramuscularly administered on days 0, 3, 14, 17, 21, 28, 31, 37, 42, and 45), as outlined in Fig. 1a. All patients were premedicated with acetaminophen and diphenhydramine prior to injection. Comprehensive safety evaluations, including physical examination, vital signs, and clinical laboratory tests (hematology, blood chemistry, urine analysis) were performed at baseline, prior to each vaccination, at predetermined time points between vaccinations, and 2 weeks after the last vaccination. Adverse events were assessed for severity and relationship to treatment, and were graded according to NCI-CTCAE version 4.0. Baseline tumor assessment was performed within 28 days prior to day 0, and restaging assessments were performed within 7 days of day 56. Objective tumor response was evaluated according to RECIST criteria version 1.1 [33, 34]. Blood samples were drawn for immune monitoring before each vaccination and two weeks after the last vaccination (days 0, 14, 28, 42, and 56). Overall survival is defined as the time from leukapheresis until death. Patients were categorized by their response (complete/partial response, stable disease, or progression) at day 56. The study was approved by the Institutional Review Board at MUSC, and was performed in accordance with the Declaration of Helsinki, Good Clinical Practice (GCP) guidelines and applicable local regulatory requirements and laws. All patients provided their written informed consent.

### DC expansion and differentiation

Peripheral blood monocyte-derived DCs were generated from peripheral blood monocyte (PBMCs)-by performing standard Ficoll-density centrifugation (GE Healthcare, Uppsala, Sweden) to isolate PBMCs from patient leukapheresed materials. PBMCs were plated in serum free AIM-V CTS media (Life Technologies, Grand Island, NY) at 2–4 × 10^8^ cells per T225 flask and allowed to adhere for 2 h in 5% CO_2_. Medium was replaced with AIM-V containing 25 ng/mL rhIL-4 (R&D Systems, Minneapolis, MN) and 800 IU/mL rhGM-CSF (Sanofi, Bridgewater, NJ) and cultured for 5–7 days at 37 °C, 5% CO_2_. DCs were cryopreserved in 10% DMSO/5% human albumin at 20 × 10^6^/mL. Prior to DC administration, a small fraction of the final DC product was used for lot release tests, which included determining DC viability, sterility, mycoplasma, endotoxin, and DC phenotypic characterization. To meet the lot release criteria the samples had to be sterile and exhibit greater than 70% viability as determined using propidium iodide (Fluka, Switzerland). The sterility testing included determination of fungal sterility using BacT/Alert Anaerobic and Aerobic bottles (Biomerieux, Durham, NC) and mycoplasma detection using commercial kit (Lonza, Switzerland). Endotoxin threshold was set at less than 5 EU/kg patient weight and determined using Endosafe®-PTS™ (Charles River Laboratories, Charleston, SC). The DC phenotypic determination for lot-release included flow-cytometry based determination of surface marker expression of CD11c, HLA-DR and CD86 (>50%), and expression for monocyte marker CD14 (<50%). All DC preparations passed lot-release criteria and were administered to patients.

### Vaccination

On the day of vaccination, cryopreserved DCs were thawed, washed in AIM-V media, counted, and resuspended to 1 × 10^6^ cells/mL and split into three equal batches. Each batch was pulsed separately with one of the following HLA-A2-binding peptides for 1 h: 20 μg/mL of the CEA altered peptide, Cap1-6D [30], 20 μg/mL of the telomerase peptide: hTERT [25–27], or 30 μg/mL of the survivin peptide [31, 32]. At the end of the incubation period, pulsed DCs were pooled, washed and re-suspended to 40 × 10^6^ cells per mL in saline. 1 × 10^7^ antigen-pulsed DCs and poly(IC:LC) were administered to the patient as previously described. DTH reactions were performed twice: 1) prior to vaccination and 2) between vaccination #3 and #4.

### Flow cytometry

PBMCs harvested at each time-point were assayed by flow cytometric analysis to determine the percentage of tetramer positive T cells. Briefly, PBMCs were first stained with fluorescent-labeled Live/Dead antibody (BioLegend, San Diego, CA) followed by blocking with 10 μg/mL human IgG [35]. PBMCs were stained with fluorescent-labeled peptide tetrameric-MHC complexes (NIH Tetramer Core Facility-Emory University) specific for the peptide epitopes used for vaccination. Additional phenotypic markers were incorporated into the staining methodology including the following: CD8 (RPA-T8), CD4 (L200), FOXP3 (259D/C7), GATA-3 (L50-823), RORγt (Q21-559), T-bet (04-46), CD44 (G44-26), CD62L (DREG-56), CD279 (EH12.1), CD80 (L307.4), CD86 (2331), CD14 (M5E2) and HLA-DR (G46-6) (BD Biosciences, San Diego, CA), and CD3 (OKT-3) (BioLegend, San Diego, CA). After staining the PBMC using standard flow cytometric methodology, cells were fixed and analyzed with a BD LSR Fortessa using BD FACSDiva 6 software (BD Biosciences, San Diego, CA). Analysis was performed using FlowJo software (TreeStar Inc, Ashland, OR).

### IFNγ ELISA/ELISPOT

The ability of PBMCs from health donors or patients to secrete IFN-γ upon recognition of specific antigen was measured via ELISA or ELISPOT. Specifically, PBMCs that were frozen at different time points post vaccination were thawed and equal number of viable cells were co-cultured overnight with T2 cells pulsed with one of the following peptides: Cap1-6D [30], hTERT [25–27], or survivin peptide [31, 32]. Control conditions were T2 cells without any peptide. Supernatant was harvested after overnight co-culturing and assayed for IFN-γ by ELISA (R&D Systems, Minneapolis, MN). Cytokine production was considered positive when IFN-γ levels are more than twofold higher after co-incubation with peptide-pulsed T2 cells compared with co-incubation with T2 cells pulsed with irrelevant antigen. Co-culture for ELISPOT (eBioscience, San Diego, CA) was conducted similarly as per manufacturer’s protocol and the antigen reactive T cells were quantified using the spots with the ELISPOT plate reader.

### Statistical considerations

This trial was designed as a pilot study for assessing feasibility and thus no power calculation was performed to justify the planned sample size (*n* = 12). Overall survival is described using Kaplan-Meier curves. Immune monitoring measures were compared using paired t-tests and Wilcoxon signed rank test were used to evaluate immune monitoring parameters. Alpha level was set at 0.05 for all hypothesis tests. For determining the antigen specific T cell response, repeated measures (% positive cells every 14 days between day 0 and day 56) were modeled using linear regression, estimated using generalized estimating equation (GEE). Time was treated as categorical and comparisons were made relative to day 0. An exchangeable correlation was assumed.

## Results

### Patients and treatment

Thirteen patients were enrolled in this single center study at MUSC. One patient (#7) developed rapidly progressive disease prior to apheresis and withdrew from the study. The baseline characteristics of the 12 patients that underwent apheresis are summarized in Table 1, Prior chemotherapy regimens, DC treatment results, and first chemotherapy after DC vaccine are outlined per patient in Table 2. Among the 12 patients who underwent apheresis, eight patients completed the study, three patients were withdrawn between days 3 and 17 due to disease progression, and one patient was withdrawn after day 17 to honor the patient’s request for hospice care.

**Table 1:** Patient characteristics

Characteristic	Study population (*n* = 12)
Median age, years (range)	64 (50–72)
Male:female	4:8
Race	
Caucasian	10
Black	2
ECOG performance status	
0	2
1	10
Disease burden at enrollment	
Locally advanced	3
Metastatic	9
Prior surgery	
Yes	1
No	11
Prior radiation^a^	
Yes	6
No	6
Number of prior systemic regimens^b^	
0	0
1	6
2	5
3	1

^a^Radiation to pancreatic bed, with concurrent capecitabine

^b^Exclusive of chemotherapy concurrent with radiation

**Table 2:** Prior chemotherapy, dc vaccine results, and first chemotherapy after dc vaccine, per patient

Patient	Disease burden at day -35	Prior chemotherapy	Response at day 56	PFS (months)	OS (months)	First treatment after DC vaccine
1	Metastatic	Gemcitabine +/- trametinib	PD	3.0	5.3	None
2	Metastatic	Gemcitabine FOLFIRINOX	Not done	2.1	2.1	None
3	Metastatic	Gemcitabine + nab-paclitaxel Capecitabine with radiation erlotinib FOLFIRINOX	Not done	1.8	2.1	None
4	Metastatic	Gemcitabine + nab-paclitaxel Capecitabine with radiation FOLFIRINOX	PD	3.0	6.3	ABC294640^a^
5	Metastatic	Gemcitabine +/- ganitumab Gemcitabine + nab-paclitaxel	SD	4.6	13.0	Gemcitabine + nab-paclitaxel
6	Metastatic	FOLFIRINOX capecitabine with radiation	PD	3.0	9.8	FOLFIRINOX
8	Locally advanced	Gemcitabine + nab-paclitaxel capecitabine with radiation	Not done	1.9	1.9	None
9	Metastatic	FOLFIRINOX gemcitabine + nab-paclitaxel	not done	2.3	2.3	None
10	Locally advanced	FOLFIRINOX capecitabine with radiation	SD	8.3	13.1	Gemcitabine + nab-paclitaxel
11	Metastatic	FOLFIRINOX capecitabine	SD	4.6	9.1	Gemcitabine + nab-paclitaxel
12	Metastatic	Gemcitabine + nab-paclitaxel + ODSH^b^	PD	3.0	10.1	None
13	Locally advanced	FOLFIRINOX Capecitabine with radiation	SD	34.3	34.3	Gemcitabine + nab-paclitaxel

^a^ABC294640 = sphingosine kinase inhibitor

^b^ODSH = 2-O, 3-O desulfated heparin

### Safety and tolerability

Study treatment was well tolerated by all patients. However, some patients experienced fatigue and/or flu-like symptoms including fever, myalgia, chills, night sweats, and/or hot flashes. When present, flu-like symptoms generally occurred within 24 h of poly(IC:LC) administration, and were self-limiting. There was one treatment interruption due to an adverse event in a patient who had treatment held on day 3 due to a grade 2 injection site reaction: this patient subsequently received all other scheduled injections. Treatment-related adverse events are summarized in Table 3.

**Table 3:** Treatment related adverse events

Adverse event	Number of patients experiencing adverse event				
	Grade 1	Grade 2	Grade 3	Grade 4	Total (%)
Fatigue	5	1	0	0	6 (50)
Hypoalbuminemia	2	4	0	0	6 (50)
Hyponatremia	6	0	0	0	6 (50)
Anemia	3	1	1	0	5 (42)
Elevated transaminases	3	2	0	0	5 (42)
Fever	4	1	0	0	5 (42)
Elevated alkaline phosphatase	2	0	2	0	4 (33)
Lymphopenia	3	1	0	0	4 (33)
Injection site reaction	3	1	0	0	4 (33)
Hypocalcemia	2	1	0	0	3 (25)
Myalgia	3	0	0	0	3 (25)
Neutropenia	3	0	0	0	3 (25)
Chills	2	0	0	0	2 (17)
Flu-like symptoms	2	0	0	0	2 (17)
Leukopenia	2	0	0	0	2 (17)
Arthritis	1	0	0	0	1 (8)
Decreased BUN	1	0	0	0	1 (8)
Hypertension	0	0	1	0	1 (8)
Hot flashes	1	0	0	0	1 (8)
Hyperbilirubinemia	1	0	0	0	1 (8)
Hypercalcemia	1	0	0	0	1 (8)
Night sweats	1	0	0	0	1 (8)
Pain	1	0	0	0	1 (8)
Pain in extremity	1	0	0	0	1 (8)

### DC preparation, characterization

Autologous DCs were generated using peripheral blood derived adherent monocytes and characterized using cell surface expression of co-stimulatory molecules (CD80, CD86), antigen presenting molecule (MHC class I, HLA-DR), and monocytic marker CD14. Greater than 70% of the DC preparation expressed CD80, CD86, and HLA-DR. As expected, the expression of CD14 was negligible and indicated that monocytes have differentiated to DC phenotype. On the day of treatment administration, three distinct DC aliquots were separately pulsed with the peptide for an hour and then mixed together before injecting intra-dermally to the patients. As depicted in the study design scheme (Fig. 1a), the DC injection was followed immediately with intramuscular injection of poly(IC:LC), a toll-like receptor 3 (TLR3) ligand in order to provide a DC maturation signal and enhance expansion of tumor infiltrating T cells. The ability of tumor antigen-pulsed DCs to activate and expand the tumor epitope-reactive T cells were measured using the peripheral blood from patients that was obtained pre- and post- DC vaccination. A representative phenotype is shown in Fig. 1b, whereas the phenotypic analysis from all patient DCs is presented in Table 4. DCs from five patients that gave consent for additional analysis, we observed that after frozen DCs were thawed and matured in vitro with poly(IC:LC) there was an increase in both IL12 and IL10 secretion to variable extent by all screened patients (i.e., # 9–13), as compared to the un-activated control DC supernatant collected overnight (Additional file 1: Figure S1).

**Table 4:** Summary of percent expression of DC phenotypic markers (*N* = 13)

DC quality parameter	Mean	Median	Range
CD14 (<25%)	4.33	0.2	(0, 29.5)
CD86 (>50%)	83.9	87.5	(60.7, 99.6)
CD11c (>50%)	97.1	99.0	(81.5, 99.6)
HLA-DR (>50%)	85.1	86.5	(71.9, 95.4)
Percent viability (>70%)	89.8	90.0	(78.9, 96.8)

### Anti-tumor activity and survival

**Fig. 2:**
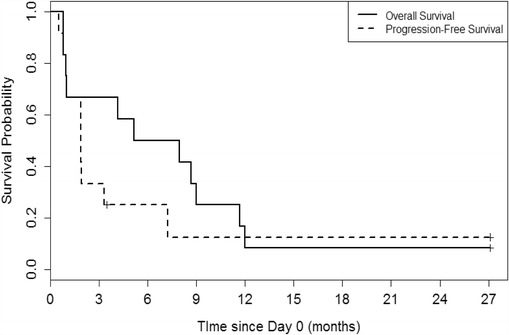
Overall and Progression-free survival. Kaplan-Meier curves show OS and PFS vs. time since leukapheresis in months

Among the eight patients who completed the study, the response at day 56 of four of the individuals was stable disease, while the other half experienced progressive disease. Of the 12 patients who underwent apheresis, the median progression free survival (PFS) was 3.0 months and the median overall survival (OS) was 7.7 months (Fig. 2). Of note, one patient (#11) initiated a non-protocol treatment prior to progression and later died. The patient’s PFS time was censored at the time of initiation of other treatment, but the time to death was included in the analysis of OS. Thus, there are 11 PFS events and there are 12 OS events (Additional file 1: Figure S2).

### Peptide-pulsed autologous DC vaccination induces epitope reactive T cells

**Fig. 3:**
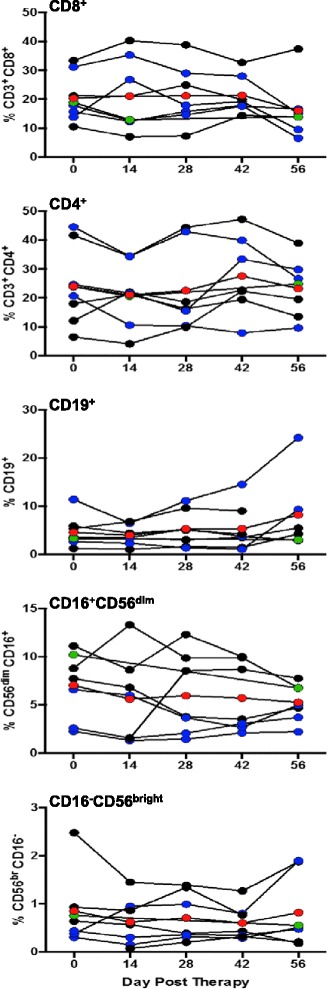
Characterization of the post-vaccination lymphocyte profile. PBMC was obtained from patients post vaccination (on days 14, 28, 42, 56). Immune cells were stained using the multiple fluorochrome-conjugated antibodies to determine the lymphocyte subsets. The data was acquired using BD FACS Aria and analyzed using FlowJo software. The percentages of cellular subsets are plotted against different time points. Each patient’s data is represented by points at each time point (blue = stable disease; black = progression) connected over time. The *green circle* represents the data from long survivor patient #13. The *red circles* represent the overall mean value at each time point

**Fig. 4:**
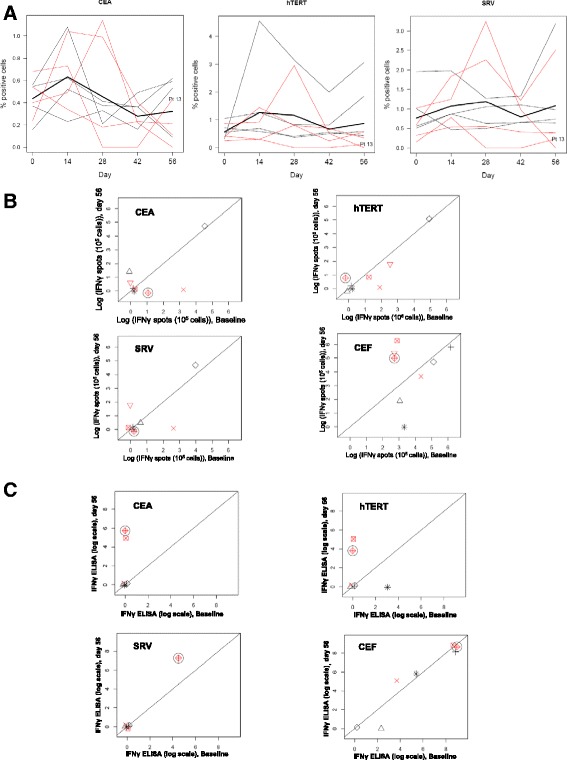
Quantitative and qualitative characterization of antigen specific T cell response post-vaccination. The durability of the antigen specific T cell response after peptide pulsed DC vaccination was determined by enumerating the difference in antigen specific T cells using the PBMCs prepared from peripheral blood drawn prior to vaccination and d56 post-vaccination. **a** The tetramer staining for the Cap1, Tert, and SRV shows the T cells reactive to these epitopes in the PBMCs for various patients at different time points. *Thin red lines* indicate patients with stable disease; thin black lines patients with progression. The line obtained from the data of long survivor patient has patient ID #13 marked next to it. Thick black line indicates fitted regression model. The data was acquired using BD FACS Aria and analyzed using FlowJo software. **b** ELISPOT assay was performed as detailed in the methods. The antigen specific re-stimulation with the tumor peptide epitope leading to secretion of the effector cytokine IFNγ was determined by quantifying the differences in ELISPOT’s between PBMC from pre vaccination (d0) *vs.* post-vaccination (d56) samples. Overnight stimulation of the pre-vaccination and post-vaccination PBMCs with the three tumor epitope peptides and CEF peptide pool was done. The CEF peptide stimulation served as positive control for the assay. Subtracting the spots seen in the unstimulated well normalized the data, and IFNγ spots were plotted at baseline vs. at day 56. Patient data is indicated by unique symbols on the plot, with red for stable disease and black for progression. Patient with long survival is indicated with black circle around his/her symbol in all of the plots. An *x* = *y* line is included to demonstrate changes from baseline (*points above the line* indicate increases; *points below the line* indicate decreases). **c** The supernatant collected after overnight re-stimulation of the pre-vaccination and post-vaccination PBMCs with the tumor epitope peptides was used to determine the IFNγ levels (pg/ml) using ELISA. The differences in pre-vaccination and post-vaccination levels were plotted. The PBMCs were also stimulated in parallel with the CEF peptide pool that served as positive control for the assay. Patient data is indicated by unique symbols on the plot, with red for stable disease and black for progression. Patient with long survival is indicated with *black circle* around his/her symbol in all of the plots. An *x* = *y* line is included to demonstrate changes from baseline (*points above the line* indicate increases; *points below the line* indicate decreases)

In order to determine if the patients (5, 6, 11, 13) with stable disease had higher frequencies of tumor antigen reactive T cells, we used the HLA-A2-peptide tetramer reagents to detect peptide-specific T cells in the peripheral blood. A comparison of pre-DC vaccination (day 0) *vs.* post-DC vaccination (day 56) showed no significant difference between the overall CD4^+^ T cells, CD8^+^ T cells, CD19^+^ B cells (CD19^+^), and NK cell (CD16^+^CD56^dim^ and CD16^+^CD56^bright^) subsets (Fig. 3, and Additional file 1: Figure S3). With reference to the differences in epitope reactive CD8^+^ T cells for all three epitopes (i.e., survivin, Cap-1, h-Tert) we noticed variable degree of expansion or contraction in the peripheral blood after each vaccination points, and no definitive correlation could be established between tumor epitope reactive T cells (as determined by tetramer staining) in the patients with stable disease (red curves) and those with progression (black curves) (Fig. 4a). We next sought to characterize if these cells secreted cytokines in an antigen specific manner. We observed that among the patients with stable disease (5, 10, 11, 13 as shown in red) exhibited a slight increase in INF-γ upon stimulation with specific antigen as measured by ELISPOT (Fig. 4b), or ELISA (Fig. 4c). The T cells activated using the CEF positive control peptide (AnaSpec, Fremont, CA) from both patients with stable disease and a few patients with progression (shown in black) exhibited a response, attesting that some viral epitope reactive T cells in patients with progression had the ability to secrete cytokine upon antigen re-stimulation. Further, detailed analysis of cell surface marker expression and transcription factors were also performed to determine the phenotype of the pre- and post- vaccinated T cells. However, we noticed that only T-bet expression was significantly increased on day 56 post-treatment as compared to T cells obtained on day 0. No significant difference was seen in the expression of other cell surface marker on either central memory (Tcm) or effector memory (Tem) cells (*data not shown*).

## Discussion

Multiple studies have shown that advanced pancreatic cancer (PC) has poor prognosis with present treatments, and indicate a need for continued efforts to find improved therapeutic approaches. A retrospective review of the dose, toxicity, and efficacy of second line gemcitabine plus nab-paclitaxel (G + Nab-P) after FOLFIRINOX in patients with metastatic and locally advanced unresectable pancreatic cancer demonstrated its modest activity and clinical benefit in advanced pancreatic cancer [36]. Results from another retrospective analysis study to determine whether cytokine-induced killer (CIK) cell-based therapy (CBT) can improve the outcomes of advanced PC appeared to imply that CBT might prolong survival in these high-risk PC patients [37]. Thus, recent advances that utilize targeting of immune checkpoint pathways, in the management of gastrointestinal malignancies are being also considered [38, 39].

Immunotherapy is transforming patient care and inducing unprecedented response rates in patients with metastatic melanoma and non-small cell lung cancer [40, 41]. Yet, these therapies remain largely ineffective in pancreatic cancer patients [42–44]. It is unclear why pancreatic cancer is poorly amenable to current immune-based therapies, but the ability to bolster an endogenous tumor-reactive T cell response may be critical to advance treatment outcome. Anti-tumor immune responses can be achieved by DC vaccines in a number of cancers, including melanoma [45–47], hepatocellular carcinoma [48], glioblastoma [49], castration-resistant prostate cancer [50, 51], renal cell carcinoma [52], acute myeloid leukemia (AML) [53], non-small cell lung cancer [54], pancreatic cancer [55, 56], and in various infectious diseases including hepatitis C virus [57] and HIV infection [58].

In this phase I trial, we sought to use a vaccination approach to generate CD8^+^ T cells in patients that could mediate immunity against tumor antigens overexpressed in pancreatic cancer. We vaccinated 12 HLA-A2^+^ patients with metastatic or advanced unresectable disease. Our vaccination strategy was comprised of peptide-loaded DCs in combination with a TLR3 adjuvant called poly-IC:LC. Moreover, the autologous DCs were pulsed with three distinct HLA-A2-restricted peptides derived from antigens expressed in pancreatic cancer: i) human telomerase reverse transcriptase (hTERT, TERT572Y), ii) carcinoembryonic antigen (CEA; Cap1-6D), and iii) survivin (SRV.A2) [25–27, 30–33]. Telomerase is a ribonucleoprotein that is responsible for RNAdependent synthesis of telomeric DNA, and is expressed in more than 90% of the pancreatic tumors. The telomerase activity is detected in pancreatic cancer but not in benign tumors [59]. Similarly, CEA is a cell surface glycoprotein and was one of the first tumor antigen to be described [60]. It is expressed in greater than 30% of pancreatic tumors [61]. Further, survivin is expressed intra-cellularly and is a member of inhibitor of apoptosis (IAP) family of proteins that is expressed in more than 80% of pancreatic tumors. The expression of survivin has been correlated with cancer cell apoptosis and in the development of human pancreatic duct cell tumors [62, 63]. Survivin was show to express more frequently in malignant tumors than in benign tumors. Given the above facts we choose the above-mentioned candidate peptides in our DC vaccine platform. This is the first phase I feasibility and safety study in pancreatic cancer patients using this approach. Not only was the treatment well tolerated, but also induced peptide-reactive CD8^+^ T cells in some patients. Among the eight patients who underwent imaging (day 56 post-treatment), four patients had stable disease and four patients progressed. The median survival was 7.7 months from date of leukapheresis, comparing favorably to the median survival of 4.2 to 4.9 months observed with second line chemotherapy for metastatic pancreas cancer [64]. We found that this vaccination approach generated antigen-specific T cells in three patients with stable disease. Although our therapy did not potentiate the frequency of endogenous CD4^+^ T cells or NK cells in the peripheral blood, it remains unknown if this regimen induced immune response in the tumor microenvironment. It is possible that DC vaccination strategies may have limited efficacy as stand-alone therapeutics. However, future studies that combine DC vaccination with other strategies, such as checkpoint modulators or T cell immunotherapy may improve the survival of patients with pancreatic cancer.

A number of reasons why DC vaccines fail to provide a successful antitumor response have been put forward, e.g., previous DC trials has been attributed to the lack of uniformity in the preparation of the DCs, maturation strategies, the antigen used for pulsing these DCs before injection, or even the antigen presenting efficiency [65]. The issue of DC exhaustion could also be responsible for high maturation signals or multiple steps of maturation leading to cell death of DCs [66, 67]. Thus, all these previous experiences with DC trials (and its failures) has led to the proposal that in order to realize the potential of DCs, it is important that multicenter phase II/III trials should be performed after standardizing the production of DC vaccines between centers [68]. The ability of DC vaccination to induce diverse neo-antigen specific T cell receptor (TCR) repertoire in terms of both TCR-β usage and clonal composition has also been implicated as one of the mechanism responsible for better tumor control [69], and needs to be considered for future immunomonitoring with the DC vaccine trials to establish its advantage over other immunotherapy regimens [70]. In addition, it has been suggested that strategy to incorporate DC-targeting via nanoparticles and combinatorial targeting of multiple human DC subsets may further improve the efficacy of DC vaccination [71]. Also, increased understanding of the DC-derived exosomes (Dex) that harbor functional MHC-peptide complexes and other immune-stimulating components [72] could enable the designing of the novel DC strategy to exploit Dex as anticancer agents. In future studies, it would be worth exploring if our vaccination approach induces neo-antigens in the tumor, which might bolster the generation of neo-antigen specific T cells in the patient, particularly when our vaccine approach is combined with checkpoint modulators.

Overall, our results herein may shed light for prospective patient selection in future immunotherapy studies. On the basis of the results of this phase I trial, we will continue developing more advanced clinical trials with this particular approach. For example, we are now conducting phase I clinical trials with our DC vaccination in HLA-A2+ patients as well as HLA-A2- patients, as the poly(IC:LC) adjuvants might enhance a plethora of antitumor specific T cells. The choice of DC administration route may impact the efficacy of vaccines. Herein, we delivered our combination therapy via an intradermal route. In our study, intradermal administration was feasible and well tolerated, warranting further development with this approach. In a related trial, we delivered this combination vaccine therapy intratumorally to the patient, in an attempt to further activate innate immune cells as well as bolster antigen-specific T cells in the patients. As there exists a fibronectin-rich shield around pancreatic tumors, it is conceivable T cells do not effectively infiltrate this malignancy. Our objective is to further define which subgroups of patients may respond to this intratumoral vaccination strategy. Our approach may contribute to further optimization of next generation DC-based vaccines for patients with advanced malignancies.

## Conclusions

Our study concludes that tumor peptide epitope pulsed autologus DC vaccination in combination with TLR ligands could be a promising approach for controlling tumor growth in pancreatic cancer patients.

## Additional file

Additional file 1: Figure S1. Cytokine secretion by DCs. Frozen DCs obtained from patients were thawed and cultured with GM-CSF/IL-4 overnight before adding poly (IC:LC) for maturation overnight. The supernatant was collected after 16 hrs. and evaluated for cytokines IL12 (upper panel), and IL10 (lower panel) as per the manufacturer’s protocol. **Figure S2.** PFS and OS details. The raw data showing details of progression free survival (PFS) and the median overall survival (OS) is presented in tabular form. **Figure S3.** Gating scheme for flow cytometry analysis. PBMC was obtained from patients post vaccination and cryopreserved cells were stained using the multiple fluorochrome-conjugated antibodies. Cells were gated based on singlets (FSC-A vs FSC-H), size (SSC vs FSC-H), a live-dead stain (L/D), and subsequently markers to determine specific cell phenotypes. A) CD3+ T cells were phenotyped for CD4 and CD8. B) CD19 B cells were identified. C) NK cells were identified based on their CD56 and CD16 expression. The data was acquired using BD FACS Aria and analyzed using FlowJo software. (PDF 351 kb)
