# Supplementary material for: Vaccination with poly(IC:LC) and peptide-pulsed autologous dendritic cells in patients with pancreatic cancer
Source: J Hematol Oncol. 2017 Apr 7;10:82. doi: 10.1186/s13045-017-0459-2 (PMC5384142; doi:10.1186/s13045-017-0459-2)
Supplement: Additional file 1: Figure S1. — Cytokine secretion by DCs. Frozen DCs obtained from patients were thawed and cultured with GM-CSF/IL-4 overnight before adding poly (IC:LC) for maturation overnight. The supernatant was collected after 16 hrs. and evaluated for cytokines IL12 (upper panel), and IL10 (lower panel) as per the manufacturer’s protocol. Figure S2. PFS and OS details. The raw data showing details of progression free survival (PFS) and the median overall survival (OS) is presented in tabular form. Figure S3. Gating scheme for flow cytometry analysis. PBMC was obtained from patients post vaccination and cryopreserved cells were stained using the multiple fluorochrome-conjugated antibodies. Cells were gated based on singlets (FSC-A vs FSC-H), size (SSC vs FSC-H), a live-dead stain (L/D), and subsequently markers to determine specific cell phenotypes. A) CD3+ T cells were phenotyped for CD4 and CD8. B) CD19 B cells were identified. C) NK cells were identified based on their CD56 and CD16 expression. The data was acquired using BD FACS Aria and analyzed using FlowJo software. (PDF 351 kb) [file 13045_2017_459_MOESM1_ESM.pdf]

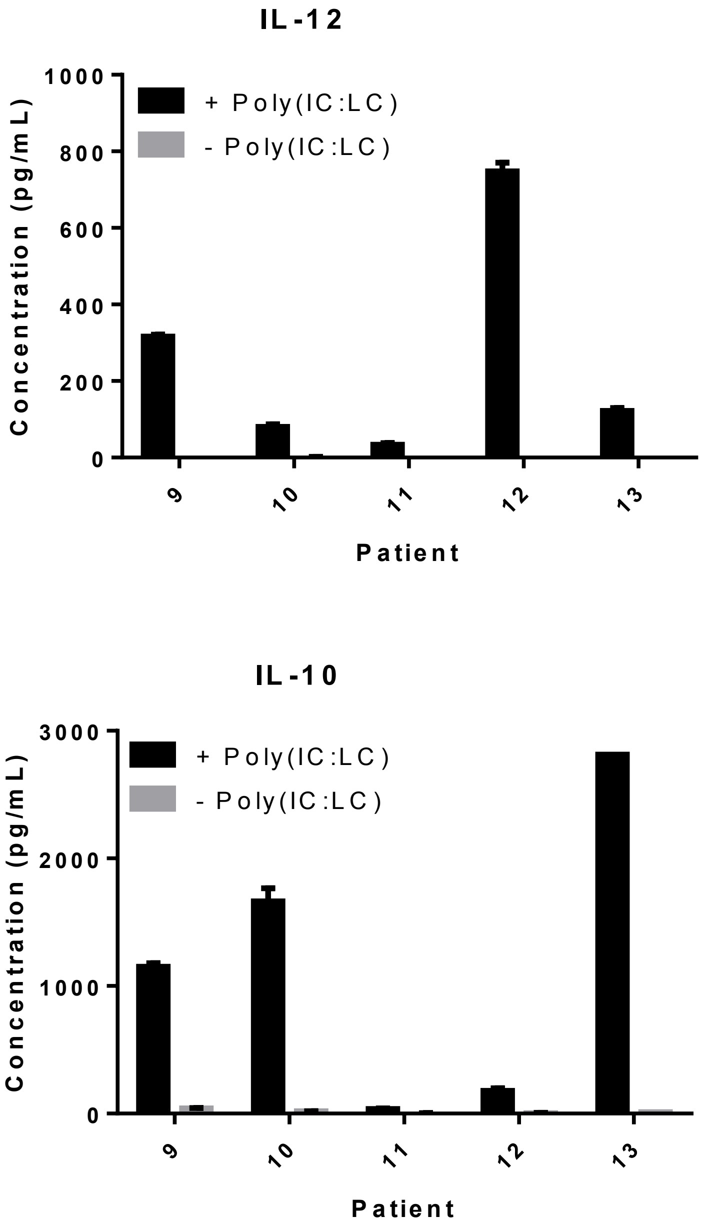

Supplementary Figure 1

Study ID*	Registration date	Baseline Scan Date	Leukapheresis Date	Day 0	Date of Progression	Date of last clinic visit	Date of first non-protocol therapy	Date of Death	PFS date	PFS event	OS date	OS event
1	3/15/12	12/19/11	2/10/12	3/15/12	5/11/12			7/18/12	5/11/12	1	7/18/12	1
2	4/25/12	2/22/12	3/22/12	4/25/12		4/30/12		5/23/12	5/23/12	1	5/23/12	1
3	5/30/12	4/11/12	4/20/12	5/30/12	6/14/12			6/23/12	6/14/12	1	6/23/12	1
4	9/26/12	8/7/12	8/22/12	9/26/12	11/21/12		12/19/12	2/27/13	11/21/12	1	2/27/13	1
5	10/26/12	10/1/12	10/26/12	12/4/12	3/13/13		3/26/13	11/19/13	3/13/13	1	11/19/13	1
6	11/1/12	10/23/12	11/9/12	12/12/12	2/6/13		2/12/13	8/29/13	2/6/13	1	8/29/13	1
8	12/12/12	11/21/12	12/13/12	1/16/13		2/1/13		2/9/13	2/9/13	1	2/9/13	1
9	2/22/13	2/18/13	3/1/13	4/10/13				5/10/13	5/10/13	1	5/10/13	1
10	6/20/13	6/20/13	6/21/13	7/24/13	2/26/14		12/18/13	7/19/14	2/26/14	1	7/19/14	1
11	6/26/13	5/29/13	7/3/13	8/6/13		1/13/14	11/18/13	4/2/14	11/18/13	0	4/2/14	1
12	7/9/13	6/18/13	7/12/13	8/14/13	10/9/13			5/10/14	10/9/13	1	5/10/14	1
13	7/30/13	7/19/13	8/1/13	9/10/13		12/2/12		5/25/16	5/25/16	1	5/25/16	1

Supplementary Figure 2

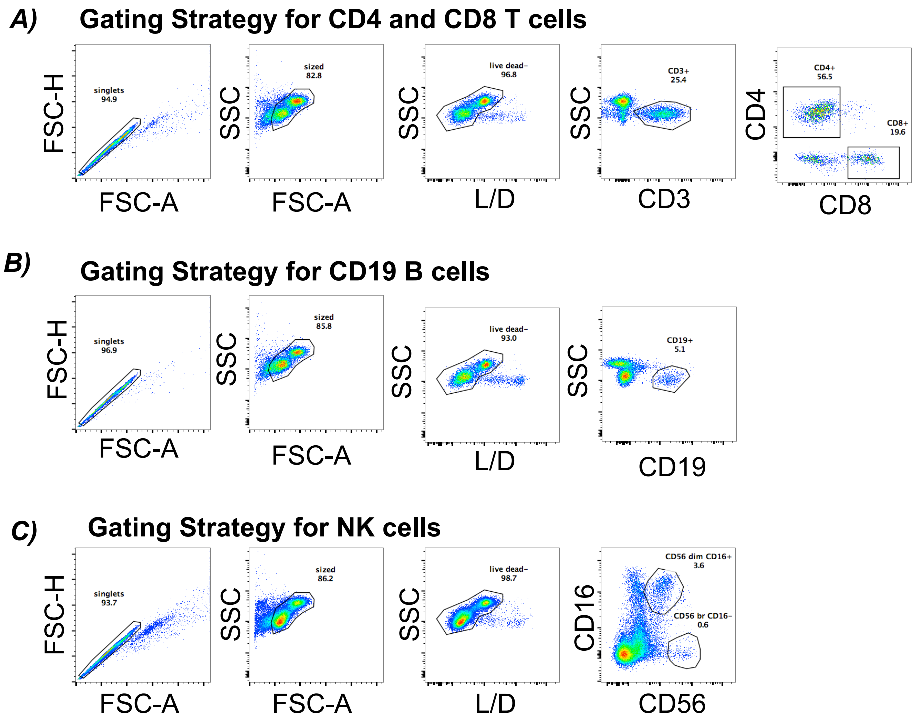

Supplementary Figure 3

Supplementary Figure Legends

***Supplementary Figure 1*: Cytokine secretion by DCs.** Frozen DCs obtained from patients were thawed and cultured with GM-CSF/IL-4 overnight before adding poly (IC:LC) for maturation overnight. The supernatant was collected after 16 hrs. and evaluated for cytokines IL12 (*upper panel*), and IL10 (*lower panel*) as per the manufacturer's protocol.

***Supplementary Figure 2*: PFS and OS details.** The raw data showing details of progression free survival (PFS) and the median overall survival (OS) is presented in tabular form.

***Supplementary Figure 3*: Gating scheme for flow cytometry analysis.** PBMC was obtained from patients post vaccination and cryopreserved cells were stained using the multiple fluorochrome-conjugated antibodies. Cells were gated based on singlets (FSC-A vs FSC-H), size (SSC vs FSC-H), a live-dead stain (L/D), and subsequently markers to determine specific cell phenotypes. **A)** $CD3^{+}$ T cells were phenotyped for CD4 and CD8. **B)** CD19 B cells were identified. **C)** NK cells were identified based on their CD56 and CD16 expression. The data was acquired using BD FACS Aria and analyzed using FlowJo software.
